# Anthropometric Characteristics and Body Composition Changes in a Five-Time Olympic Champion in Greco-Roman Wrestling: A Longitudinal Case Study Towards the Paris 2024 Olympic Games

**DOI:** 10.3390/jfmk10020176

**Published:** 2025-05-15

**Authors:** Wiliam Carvajal-Veitía, Carlos Abraham Herrera-Amante, Rodrigo Yáñez-Sepúlveda, Vladimir Gainza-Pérez, Yanell Deturnell-Campos, Carlos Cristi-Montero, Guillermo Cortés-Roco, César Octavio Ramos-García

**Affiliations:** 1 Subdirectorate of Teaching and Research, Institute of Sports Medicine (IMD), Havana 10800, Cuba; wiliamcarvajal790@gmail.com; 2 Ibero-American Network of Researchers in Applied Anthropometry (RIBA2), 04120 Almería, Spain; 3 Nutritional Assessment and Nutritional Care Laboratory (LECEN), Division of Health Sciences, Tonalá University Center, University of Guadalajara (UdeG), Tonalá 45425, Mexico; 4 Faculty of Education and Social Sciences, Universidad Andres Bello, Viña del Mar 2520000, Chile; rodrigo.yanez.s@unab.cl; 5 Subdirectorate of Medical Control, Institute of Sports Medicine (IMD), Havana 10800, Cuba; prefimed@gmail.com (V.G.-P.); yanell201074@gmail.com (Y.D.-C.); 6 IRyS Group, Physical Education School, Pontificia Universidad Católica de Valparaíso, Valparaíso 2530388, Chile; carlos.cristi.montero@gmail.com; 7 Facultad de Ciencias de la Vida, Universidad Viña del Mar, Viña del Mar 2520000, Chile; guillermo.cortes@uvm.cl

**Keywords:** wrestling, elite athlete, anthropometry, body composition, somatotype, sports nutritional sciences, athletic performance

## Abstract

**Purpose**: This case study examines the anthropometric characteristics and body composition changes of a 41-year-old Cuban Greco-Roman 130 kg wrestler, a five-time Olympic gold medalist (2008–2024). To optimize his preparation for the Paris 2024 Olympic Games, another athlete participated in the qualifying process, allowing him to train without competition gear. **Methods**: The study monitored changes in body composition using anthropometry and bioelectrical impedance analysis (BIA) at three key time points in 2024: January, June, and July. The final assessment occurred 25 days before the Olympic event, coinciding with the final phase of his preparation. **Results**: The analysis revealed a significant reduction in total body mass, from 150 kg in January to 138.5 kg in July, with fat mass decreasing from 37.06 kg (24.11%) to 29.7 kg (21.5%). Muscle mass decreased slightly (77.41 kg to 72.3 kg), while bone mass remained stable. The somatotype classification was endomorphic–mesomorphic at all assessments, with slight shifts in its components (4.6–10.4–0.1 in January to 4.4–10.3–0.1 in July), reflecting an improved muscle–fat ratio. Notably, hydration levels and cellular integrity remained stable, as indicated by BIVA analysis. **Conclusions**: This study provides insight into the anthropometric characteristics and body composition of an elite Greco-Roman wrestler, as well as the changes observed during his preparation for his final Olympic participation. These data serve as a valuable reference for wrestlers and sports professionals, highlighting the physical profile of one of the most emblematic figures in Olympic history.

## 1. Introduction

Greco-Roman wrestling is a weight-classified Olympic event that requires exceptional upper-body strength, motor control, and technical precision because all action is confined above the waist. Athletic performance relies on a combination of explosive, high-intensity efforts—such as throws and lifts—and sustained aerobic capacity to endure two physically demanding rounds [1,2,3]. A common and controversial practice in this sport is weight cutting, which involves a rapid reduction in body mass (BM) to meet the demands of competition [4,5]. Techniques such as dehydration, caloric restriction, and fluid deprivation pose significant physiological and psychological challenges, including glycogen depletion, hormonal imbalances (e.g., cortisol and testosterone), and increased psychological stress, which can impair strength, endurance, and recovery [6,7,8,9,10,11,12,13,14].

When body mass loss exceeds 5%, the negative impact on athletic performance becomes more pronounced [15,16]. In contrast, gradual weight management strategies, such as those recommended by the American College of Sports Medicine, aim to minimize these risks by preserving muscle mass and ensuring adequate hydration [17]. Accordingly, an assessment of body composition is critical in evaluating the effects of different weight management strategies on both performance and athlete health.

Wrestlers often begin reducing BM at least two weeks prior to competition [18,19,20]. However, longitudinal data documenting prolonged periods of significant BM reduction—greater than 10%—remain scarce. Notably, Kordi et al. [21] found that 5% of 198 Iranian wrestlers lost more than 10% of their BM prior to competition, while Zhong et al. [22] reported losses of up to 17.5% in martial artists within a 29-day window.

Although wrestling is one of the most studied sports in terms of weight management and BM loss [18,23,24,25,26], longitudinal studies evaluating the effects of prolonged BM reduction on anthropometric profiles, fractional body composition, and bioelectrical impedance vector analysis (BIVA) are still lacking, despite the widespread acceptance of these methods in applied sports science [27,28,29,30].

The need for such research is particularly pressing in the case of heavyweight wrestlers, whose unique physiological characteristics and competitive demands are underrepresented in the literature. This study addresses this gap by investigating the effects of an extended weight management intervention in an elite athlete: a five-time Olympic heavyweight Greco-Roman wrestler, aged 41, who returned to competition at the Paris 2024 Games after an extended hiatus following Tokyo 2021. Over an eight-month preparation period, the athlete achieved a body mass reduction of over 10% while maintaining an exceptional 31-year career in elite sport. Through a multidimensional approach—including whole-body, tissue, cellular, and molecular assessments—this study provides novel insights into the physiological adaptations associated with prolonged weight management in elite sport and offers practical implications for evidence-based coaching and health management of athletes in heavyweight wrestling. In addition, the study describes the anthropometric and morphofunctional characteristics of the athlete, including body composition, somatotype, and human proportionality scores, that make him unique and distinguish him from others in his category.

## 2. Materials and Methods

### 2.1. Athlete and Case Study Background

A case study in which the athlete was a Cuban Olympic wrestler, aged 41.4 decimal years, with a stature of 194.4 cm. Now retired from elite competition, he competed in the 130 kg Greco-Roman wrestling category at the Paris 2024 Olympic Games. He previously won gold medals at the 2008 Summer Olympics in Beijing, 2012 in London, 2016 in Rio de Janeiro, and 2020 in Tokyo.

The athlete took a break after the Tokyo Olympics until January 2024, when he began a six-month preparation period. He did not compete for the Olympic qualification in the 130 kg category for the Paris Games in 2024; instead, another athlete secured the qualification at the 2023 World Championships in Belgrade [31]. This strategy allowed the Athlete to prepare for the Paris 2024 Games without competitive demands, given his age.

The athlete has read, approved, and provided written consent for this publication, which meets the ethical standards for case studies. The procedure followed the ethical standards of the Institute of Sports Medicine (IMD) Ethics Committee on Human Experimentation (code: CEI-IMD-01-11-2023) and complied with the 2013 updated Declaration of Helsinki [32]. This case study adheres to the CARE (for CAse RE-ports) guidelines [33].

As part of the confidentiality protocols, data on body mass loss during the last 25 days before the competition, when 8.5 kg (6.1%) remained to reach the competition weight, will not be disclosed. Protection of this information is an integral part of the procedures established in the Kinanthropometry Laboratory of the Institute of Sports Medicine.

### 2.2. Nutritional Intervention Strategy

The body mass management strategy, designed according to the recommendations of Reale et al. [34], was structured in three progressive phases. The goal was to achieve controlled fat reduction, preserve muscle mass, and maintain cellular integrity to ensure that the athlete reached peak condition to compete in the Paris 2024 Olympic Games after a hiatus since Tokyo 2021.

The first phase, known as the initial stabilization phase, ran from January 9 to June 6, 2024, and aimed to restore the athlete’s physical readiness without immediate competitive pressure. The 9 January and 6 June assessments used anthropometry and impedance methods to monitor the athlete’s condition. This phase focused on a controlled energy balance with an adjusted macronutrient intake: protein (1.6–2 g/kg/day), carbohydrates distributed throughout training sessions (4–6 g/kg/day), and healthy fats accounting for 25–30% of total caloric intake. Hydration protocols were optimized to ensure cellular functionality and water balance stability.

The second phase, termed the active strategy phase, ran from 6 June to 11 July 2024, and implemented progressively aggressive nutritional strategies to induce significant body mass loss. Protein intake was increased to 2–2.2 g/kg/day, carbohydrates were adjusted to 3.5–5 g/kg/day, and fats were reduced to 20–25% of total caloric intake. A final assessment on July 11th evaluated the adjustments made, with hydration remaining a priority to maintain cell quality throughout the body mass loss process.

Although the third phase, the cutting phase, was not specifically evaluated, the athlete successfully reached the competition weight of 130 kg on August 5 and 6, following the same principles established by Reale et al. [34]. This phase followed progressive caloric deficit strategies, maintaining consistency with the nutritional and hydration framework of the previous phases to ensure readiness for competition.

**Figure 1:**
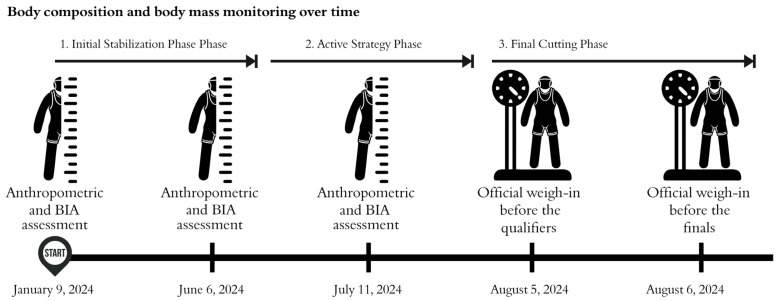
Flowchart of the experimental design for the weight management strategy in preparation for Paris 2024.

The experimental design included training sessions at high performance camps in Bulgaria and Croatia, with regular evaluations at the Anthropometry Laboratory of the Institute of Sports Medicine in Havana, under the supervision of an ISAK-certified specialist. This meticulous approach to monitoring and progressive adjustments at each stage addressed a critical gap in the literature on weight management in combat sports. Figure 1 illustrates the complete flow of this experimental design, detailing the sequence and objectives of each phase.

**Figure 2:**
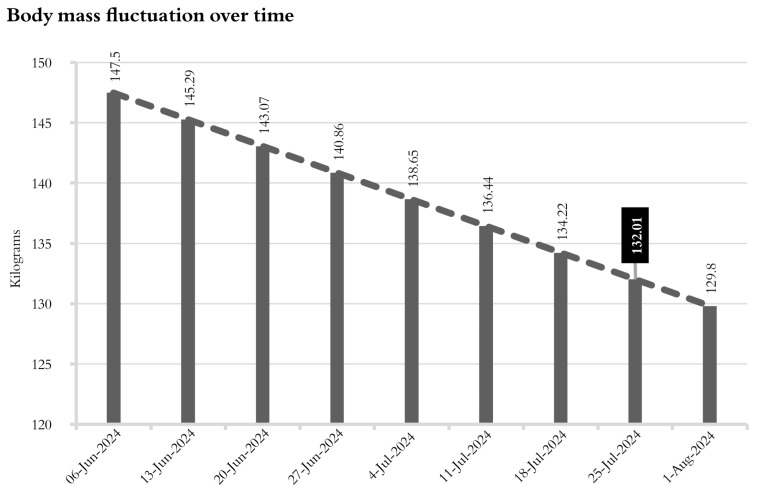
Estimated body mass on the measurement dates. The estimated body mass was reached on 25 July 2024.

At the beginning of the athlete’s preparation process (9 January 2024), his minimum wrestling weight (MWW) was certified at 132.8 kg. The projection for reaching the MWW was set by the American College of Sports Medicine [17]: a loss of 1.5% BM per week starting on 6 June (Figure 2).

### 2.3. Experimental Procedures, Measurements, and Data Analysis

Body composition was assessed using two different methods: (i) anthropometry and (ii) bioelectrical impedance analysis (BIA). All data from the developmental assessment were presented at three points prior to the Olympic event (January, June, and July). The final evaluation was performed 25 days before his participation in the Paris 2024 Olympic Games, coinciding with the final stage of his preparation.

### 2.4. Anthropometric Profile: Body Composition, Somatotype, Indices, and Phantom Proportionality

**Figure 3:**
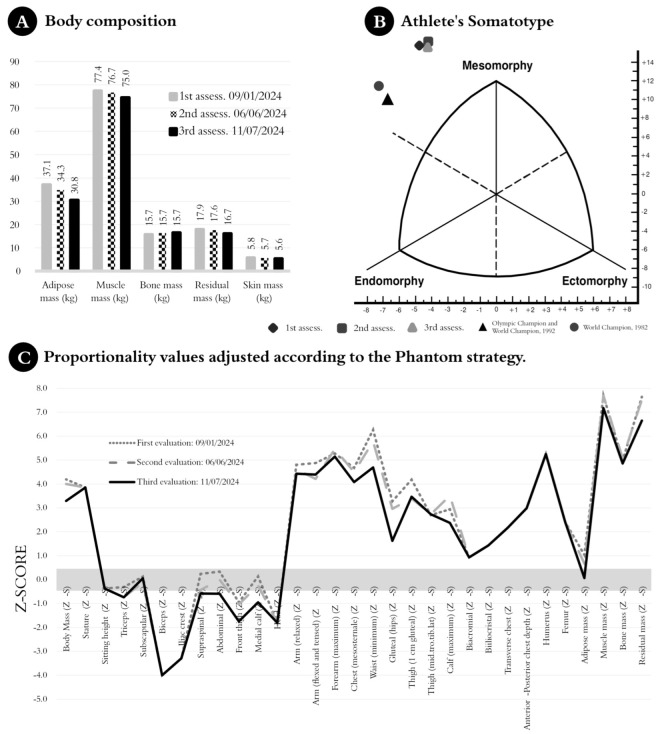
Evolution of body composition, somatotype, and human proportionality based on anthropometric variables (anthropometric profile) across three key evaluations conducted on 2024: 9 January, 6 June, and 11 July, prior to the Paris 2024 Olympic Games (charts (**A**)–(**C**)).

The assessments were performed by the same level III anthropometrist according to the protocols established by the International Society for the Advancement of Kinanthropometry (ISAK) standards [35]. During 2024, 27 anthropometric variables were measured based on the phantom stratagem [36], as shown in Figure 3. Analyses were performed using Holtain instruments (Holtain Ltd., Crymych, UK). The technical measurement error was less than 5% for skinfolds and less than 1% for other anthropometric variables. Body composition was determined using the five-fold fractionation method of body mass [27], which includes an estimation of fat mass. However, fat mass was also estimated using Lohman’s equation [37], which has been validated for the calculation of minimum wrestling weight (MWW = fat-free mass/0.87) in wrestlers [17]. A reference value of 13% was used for MWW as this was the lowest fat percentage achieved by this athlete during his peak performance at the London 2012 Games. Somatotype was determined using the Heath–Carter anthropometric method [38].

### 2.5. Body Composition Assessed by BIA

Body composition was assessed by BIA as a complementary method using the Seca medical Body Composition Analyzer (mBCA) 214/215 (Seca GmbH and Co., KG, Hamburg, Germany). The indicators evaluated included fat mass and fat-free mass indices, phase angle, total body water, extracellular and intracellular water, resistance, reactance, and qualitative indicators such as bioelectrical impedance vector analysis (BIVA) and body composition chart (BCC).

The Seca mBCA device used in this study is considered a reliable instrument, having been validated against established reference methods, including the four-compartment model (4C), deuterium dilution (D2O), and sodium bromide dilution (NaBr). Empirical evidence has consistently demonstrated correlations greater than 95% for critical parameters such as fat-free mass and total body water, underscoring its accuracy and applicability in tracking anthropometric and compositional changes [39,40,41].

From a reliability perspective, the device exhibits minimal intra-device variability, ensuring consistent measurements when performed under standardized conditions [42]. In addition, the study protocol included an ISAK-certified operator, which minimized intra-operator variability and increased the reproducibility of results.

### 2.6. Analysis Approach

The anthropometric and bioimpedance results in this case study were expressed as percentage change over the measurement series. Bar charts, somatochart, and line graphs were used to illustrate trends in body mass fractionation, somatotype, and proportionality profile. Somatotype analysis included trend comparisons with two reference groups: world championship medalists and Olympic athletes in the same competitive category from Cuba.

## 3. Results

### 3.1. Anthropometric Profile: Body Composition, Somatotype, Indexes, and Phantom Proportionality

The monitoring of the athlete’s anthropometric profile is shown in Table 1. From the first to the last evaluation, absolute and relative values of body mass, fat mass, bone mass, body fat (Lohman), endomorphy, WHR, AMR, BMI, and ∑6SKF decreased more significantly than muscle mass, FFM (Lohman), mesomorphy, and ∑4CG. Residual mass, ectomorphy, bone mass, thorax, HBBI, HBiiL, and BBI indices remained unchanged.

In the first 150 days, from January to July, the changes were less pronounced than those observed in the following 36 days, from 6 June to 24 July 2024, 25 days before the tournament.

With the most significant change in body mass, from 147.5 to 138.5 kg, the athlete lost 6.1% of BM, 10% of adipose tissue mass, and only 2.2% of muscle mass. Lipid mass decreased by 16.1%, whereas FFM decreased by only 3.29%. The sum of skinfold thicknesses (∑6SKF) changed by 13.5%, while corrected girths (∑4CG) decreased by only 1%.

### 3.2. Anthropometric Parameters: Body Composition, Somatotype, and Proportionality

Figure 3A shows a significant reduction in total body mass, from 150 kg in January to 138.5 kg in July. In terms of absolute body mass fractionation, this change was most pronounced in fat mass (37.06 kg to 29.7 kg), while muscle mass showed a less pronounced reduction (77.41 kg to 75.0 kg). Other compartments decreased slightly.

The athlete’s somatotype was categorized as endomorphic mesomorph (4.9–10.4–0.1), indicating a pronounced musculoskeletal development over relative adiposity. By July, this profile had shifted to a lower endomorph (4.3–10.1–0.1), as shown in Table 1. Compared to World Championship medalists from the 1980s and 1990s, this athlete had greater mesomorphy and lower relative adiposity (Figure 3B).

Figure 3C shows the changes in body proportionality using the phantom strategy, expressed as z-scores. The most pronounced changes occurred in waist girth (minimum, Z-S), from 6.3 to 4.7. Skinfold thickness measurements such as triceps (Z-S) and subscapular (Z-S) also showed slight reductions, with triceps (Z-S) decreasing most significantly from −0.3 to −0.7. BM (Z-S) decreased from 4.2 in January to 3.3 in July. Fat mass (Z-S) decreased from 7.7 to 7.2, while muscle mass (Z-S) remained stable at high values (~5.0).

### 3.3. Body Composition Assessed by BIA

Table 2 provides a detailed summary of the evolution of body composition at the cellular and molecular levels using bioelectrical impedance analysis (BIA). At the cellular level, phase angle (PA) showed a slight decrease from 6.1° in January to 5.6° in June and July. The absolute increase in TBW was most pronounced from January to June, coinciding with greater increases in ICW and IWC/EWC than in ECW, resulting in a relative decrease in ECW/ICW. During the June–July rapid loss period, changes in TBW (0.0 L), extracellular water (+0.3 L), and intracellular water (−1.9 L) were not substantial. The resistance decreased continuously, but the reactance remained stable since June.

At the molecular level, the fat mass index (FMI) decreased significantly by 40.1%, while the fat-free mass index (FFMI) initially increased before decreasing (27.01 to 29.45 kg/Ht^2^).

**Figure 4:**
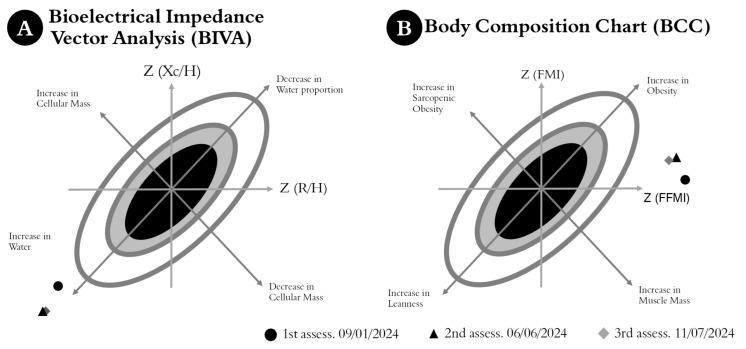
Analysis of the evolution of the bioelectrical impedance vector (BIVA) and body composition (BCC) in three key evaluations conducted in 2024, prior to the athlete’s participation in the Paris 2024 Olympic Games (**A**,**B**). Dark markers represent the athlete’s body composition at the cellular and molecular levels: black circles for January, black triangles for June, and gray diamonds for July 2024.

Figure 4A illustrates the evolution of the Bioelectrical Impedance Vector Analysis (BIVA), highlighting changes in the athlete’s cellular and molecular state throughout the evaluated period. A high proportion of body water relative to cell count was observed from the first measurement. Figure 4B shows an FFMI above the population average, with migration reflecting a decrease in FMI, while FFMI remained stable.

## 4. Discussion

### 4.1. Anthropometric Characteristics of Anthropometric Profile

This study represents a significant advancement in sports science as no previous research has specifically characterized the anthropometric profile of wrestlers competing in the 130 kg category across wrestling styles. Most existing studies tend to group super-heavyweight wrestlers with lighter categories or exclude them altogether to avoid increasing morphological variability, thus failing to account for their distinct structural and functional characteristics [43,44,45]. Consequently, the findings presented in this study offer a new perspective on performance analysis and talent identification in Greco-Roman wrestling.

Body composition and somatotype are fundamental pillars in determining competitive success in this discipline [46,47,48]. The elite wrestler analyzed in this study exhibits strong mesomorphic dominance, characterized by a high fat-free mass (FFM) and optimized structural proportions, establishing a new benchmark for evaluating athletes in the super-heavyweight category.

The analyzed wrestler showed superior values compared to the Cuban average for the same competitive category in several anthropometric parameters [48]. He demonstrated notable advantages in stature (194.4 cm vs. 188.0 cm), mesomorphy (10.1 vs. 7.0), the sum of skinfold thickness (99.6 mm vs. 79.6 mm), and body fat percentage (19.5% vs. 11.7%), reinforcing his structural profile optimized for performance. However, in terms of active body substance index (1.52 vs. 1.59 g/cm^3^), the wrestler showed slightly lower values than the category average, which could indicate differences in the distribution of functional mass. In terms of somatotype, the subject was classified as endomorphic–mesomorphic (4.3–10.1–0.1), similar to the Cuban average for the 130 kg category (3.0–7.0–2.4), but with a greater predominance of endomorphy and mesomorphy. This suggests an evolutionary trend towards a more compact and structurally efficient morphology, adapted to the increasing physical demands of Greco-Roman wrestling.

In terms of somatotype evolution, the current Cuban champion shows a marked difference from previous Cuban medalists, as shown in Figure 3B. He is significantly more mesomorphic than a world champion who competed in the 120+ kg category in the 1980s and significantly more mesomorphic than another Olympic and world medalist who competed in the 100 and 120 kg categories in the late 1990s. This trend suggests that the Cuban 130 kg wrestler has evolved toward a more compact somatotype, likely as an adaptive response to the increasing physical demands of the sport.

While the primary focus of this study is on the individual anthropometric characteristics of the wrestler, a comparison with Azerbaijani wrestlers provides additional context for evaluating his physical advantages. Rahmani et al. [47] reported that Azerbaijani wrestlers in this category had an average stature of 183.83 cm, a BMI of 32.9 kg/m^2^, a %BF of 14.8, an FFM of 102.86 kg, and somatotype values of 3.13–8.76–0.6. In contrast, the Cuban athlete exceeded these parameters with a stature of 194.4 cm, a BMI of 36.6 kg/m^2^, an FFM of 111.5 kg, and somatotype values of 4.3–10.1–0.1. Due to methodological differences in body fat measurement, %BF values cannot be directly compared, but the marked variation in somatotype and FFM reinforces the structural advantages of the Cuban wrestler.

This study fills a critical gap by providing a focused examination of an elite competitor at the highest level of the sport. While direct comparisons remain limited, the reference to Azerbaijani wrestlers is highly relevant as Azerbaijan consistently produces world-class athletes in Greco-Roman wrestling, solidifying its status as a dominant force in the discipline.

Regarding anthropometric indices, although previous studies—such as the research by Škugor et al. [43] on Croatian wrestlers—have shown that anthropometric indices do not show significant differences between medalists and non-medalists, the results of this study should not be disregarded in talent selection. On the contrary, these indices remain essential for identifying structural patterns associated with performance outcomes, allowing coaches to make strategic decisions regarding athlete development.

An example of this can be seen by examining the proportionality profile between the Polish wrestlers and the subject of the study (Figure 3C). The femur width, humerus width, and flexed arm girth of the Cuban athlete reflected greater skeletal robustness and muscular development (Z-S > 0 and Z-S < 5). In contrast, Polish wrestlers had more balanced proportions across weight classes (Z-S from 1.76 to 2.69) [45].

When analyzing the proportionality profiles of the subscapular, triceps, biceps, and calf skinfolds, the Cuban athlete presented higher skinfold thickness values, indicating greater energy reserves (Z-S > −4 and Z-S < 0). Polish wrestlers, on the other hand, maintained lower values, emphasizing a leaner structure adapted to their performance strategies (Z-S from −1.82 to −2.19). These results confirm how super-heavyweight athletes develop specific anthropometric adaptations, differentiating the structural advantages of the Cuban athlete in biomechanical leverage from his Polish counterparts.

An innovative application within this study is the estimation of minimal wrestling weight (MWW) based on a 13% body fat (BF) threshold, which provides a more realistic reference for super-heavyweight wrestlers. Contrary to the 5% BF threshold recommended by the American College of Sports Medicine [17,49], which applies mainly to light- and medium-weight categories, long-term analyses of more than 500 combat sports athletes (wrestling, judo, boxing, and taekwondo) at the Cuban Institute of Sports Medicine (IMD) have shown that this lower threshold is unattainable for heavier athletes, even under optimal conditions.

Although a limitation of this research, the study lacks body composition data at competition weight as the athlete underwent an additional 8.5 kg reduction in the last 25 days prior to the event. However, his initial %BF of 19.5 provided sufficient physiological reserves to complete the body mass loss process without compromising muscle integrity. In contrast, in lower-weight-class wrestlers, FFM becomes the primary limiting factor during body mass loss. In such cases, athletes often experience significant muscle wasting during the acute body mass reduction phase, particularly in the days leading up to competition [43,50].

This finding suggests that super-heavyweight wrestlers may exhibit distinct patterns of body composition management, particularly in their ability to maintain higher initial fat percentages while maintaining functional muscle mass throughout the body mass loss process. Unlike their lighter counterparts, who often approach critical physiological thresholds in terms of fat-free mass availability, athletes in the 130 kg category appear to possess greater metabolic flexibility, allowing them to progressively reduce body mass while maintaining performance capacity. This adaptation may be essential for maintaining competitive readiness in the super-heavyweight divisions, where absolute strength, structural integrity, and endurance play a critical role in performance outcomes.

### 4.2. Analysis of Body Mass Monitoring

The process of body mass reduction in this elite athlete provides critical insights into the physiological and structural adaptations required for high-performance competition. One of the key findings of this study is the athlete’s ability to achieve a controlled and balanced reduction of 6.1% body mass over 36 days, primarily through targeted fat loss while maintaining muscle mass and phase angle (PhA). These results are consistent with those of Reale et al. [37] and confirm that gradual and structured body mass loss strategies are essential in elite sports.

A key methodological innovation in this study was the use of non-traditional anthropometric techniques, such as corrected girth and five-way fractionation of body mass, which provided a more detailed perspective on composition shifts. In contrast to conventional methods, this approach allowed precise differentiation between reductions in adipose tissue and muscle mass, providing a deeper understanding of the physiological adaptations of the athlete during body mass loss.

Fluid balance played a fundamental role in this process. Previous studies using dilution techniques have linked fluctuations in the ICW/ECW ratio to cellular damage, inflammation, and dehydration [31]. In this case, however, the athlete exhibited acute hyperhydration due to increased fluid intake and optimized sodium levels during the rehydration phase. This strategy, combined with a controlled reduction in sodium intake in the days prior to competition, is consistent with the hydration protocols recommended by Reale et al. [37].

The stability of phase angle and reactance throughout this body mass loss process underscores the effectiveness of hydration and nutritional management in maintaining cellular integrity, confirming the findings of Campa et al. [29]. The absence of significant changes in intracellular and extracellular water ratios, bioelectrical impedance vector analysis (BIVA), and body cell mass further highlights the athlete’s resilience to fluid balance shifts—in contrast to amateur boxers who often experience dehydration effects with similar body mass losses [28].

Regarding the relationship between phase angle, intracellular water (ICW) and ECW/ICW ratio, this study contradicts previous findings by Marini et al. [51] and Campa et al. [52]. Marini et al. [51] reported a positive correlation between PhA and ICW (r = 0.327, *p* < 0.001) and a negative correlation with ECW/ICW (r = −0.493, *p* < 0.001), whereas Campa et al. [52] found that PhA was positively correlated with TBW and ICW (r = 0.458, *p* < 0.01 and r = 0.564, *p* < 0.01, respectively) and negatively correlated with ECW/ICW (r = −0.436, *p* < 0.01). In contrast, ICW and TBW increased in this study, while PhA decreased from 6.1° to 5.6°, reversing previous trends.

Despite this discrepancy, the BIVA vector shift and the increase in the ICW/ECW ratio from 1.22 to 1.39 suggest improved cellular stability and metabolic efficiency. These observations are consistent with findings in Chinese and European athletic populations. Zhang et al. [53] demonstrated that ICW/ECW values between 1.35 and 1.45 were indicative of optimal cellular stability and performance, supporting the physiological adaptations observed in this study.

Similarly, Canda et al. [54] studied Spanish athletes and concluded that moderate fluctuations in PhA could be associated with hydration adaptations without compromising cellular integrity. Their research found a mean PhA of 7.3° in male athletes, whereas the PhA values recorded in this study ranged from 6.1° to 5.6°, suggesting a sport-specific adaptation to body mass loss rather than a decline in cellular function.

These findings support the hypothesis that super-heavyweight athletes may exhibit a differential regulation of cellular conductivity, consistent with previous observations by Marini et al. [51] and Campa et al. [52], where bioelectrical parameters varied according to body composition and body mass loss history. In this context, the shift of the BIVA vector towards higher total water content, together with the sustained increase in ICW, confirms that the fluid management strategy implemented in this athlete favored optimal fluid redistribution without compromising muscle function.

In terms of phase angle interpretation, values between 5° and 7° indicate good cell structure and membrane integrity, while lower values are associated with fluid accumulation and a loss of membrane integrity, and higher values are associated with dehydration [55]. The range of 6.1° to 5.6° recorded in this study falls within these physiological parameters. However, comparisons with reference values established by Campa et al. [56] for Italian athletes using the BIA 101, Akern (Florence, Italy), are not applicable to this Greco-Roman wrestler due to technological and methodological differences.

Although phase angle estimates do not require biological assumptions [30], research has shown that raw impedance parameters—resistance, reactance, and phase angle at 50 kHz—vary between devices due to technical factors [57]. As a result, data from this study can only be compared to results obtained using similar bioimpedance technologies, specifically those that incorporate mBCA formulas.

In addition to tracking body mass loss, phase angle monitoring has been shown to be a valuable tool in assessing somatic maturation over the course of a competitive season [51]. Additionally, PhA is positively correlated with vertical jump performance [58], a critical variable influencing the competitive success of Greco-Roman wrestlers [1,46].

In conclusion, it is important to emphasize that the case of this athlete demonstrates the benefits of structured preparation without exposure to competitive stress and body mass cycling, a strategy that may have optimized his performance and preserved his metabolic functionality. As emphasized by Lebron et al. [59] in their review on physiological disturbances in combat sports, repetitive cycles of body mass loss can lead to dysfunctions in insulin and leptin regulation, negatively affecting the energy efficiency of athletes. The training plan allowed this athlete to minimize cumulative metabolic stress, ensuring a more efficient adaptation during the Olympic qualifying period.

In addition, given his 41 years of decimal age and extensive competitive career, avoiding extreme body mass fluctuations may have reduced the impact of endocrine alterations that often affect aging athletes. Lebron et al. [59] emphasized that aggressive body mass reduction processes may impair post-event recovery and increase the risk of long-term metabolic dysfunction. The strategy adopted by the coaching staff in this case not only optimized physical preparation but also preserved metabolic homeostasis, reflecting a comprehensive approach grounded in both professional experience and scientific evidence to support the sustainability of long-term performance.

### 4.3. Limitations

One of the major limitations of this study is the lack of body composition data at competition weight as the athlete underwent an additional body mass reduction in the final days before the event. This prevents an accurate assessment of how the body mass reduction may have affected muscle integrity, hydration levels, and overall physiological adaptations. Additionally, while the anthropometric profile of a super-heavyweight wrestler has been thoroughly characterized, the findings are based on a single elite athlete, limiting generalizability to the broader population of wrestlers in this category. Methodological differences in body fat measurement between studies also challenge direct comparisons with international competitors. Despite these limitations, the study fills a critical gap by providing a reference framework for talent identification and performance optimization in the 130 kg Greco-Roman wrestling category.

Additionally, another limitation of this study is the lack of psychological assessment, despite the athlete’s significant motivation in his pursuit of a fifth Olympic gold medal. The literature highlights that mental advantage, motivational factors, and psychological responses to body mass change are often overlooked in studies of wrestlers [60]. Given the significant mental and emotional demands placed on elite athletes, future research could explore the psychological component of body mass management in Greco-Roman wrestling to better understand its influence on performance.

### 4.4. Implications for Coaches and Practitioners

This study demonstrates the usefulness of non-traditional methods in the assessment and management of body mass in super-heavyweight wrestlers, highlighting anthropometric fractionation, corrected girths, and bioelectrical impedance vector analysis (BIVA) as essential tools for accurate athlete monitoring. Fractionation enables differentiation of body compartments, allowing for adjustment of body mass reduction without compromising functional muscle mass, while corrected girths facilitate the assessment of muscle structure in key areas for performance. In addition, BIVA provides insight into cellular stability and fluid balance, ensuring that body mass adjustments do not compromise metabolic function. For coaches, the integration of these techniques enhances athletic planning, enabling more precise training and nutritional adjustments while ensuring that the body mass loss process maintains the athlete’s competitive ability.

One of the most valuable tools for coaches is the monitoring of phase angle (PhA) as an indicator of cellular integrity and hydration status. While PhA has been extensively studied in endurance and power sports [51,56,58], its application in combat sports remains underutilized. In this study, PhA remained relatively stable despite body mass loss, highlighting the effectiveness of proper hydration and nutritional protocols. Coaches should incorporate PhA tracking into athlete assessments to ensure that body mass loss does not compromise physiological resilience.

In addition, coaches can use intracellular and extracellular water (ICW/ECW) monitoring to assess hydration levels and fluid balance [28,52]. The maintenance of ECW/ICW ratios without significant changes in body composition suggests that careful hydration strategies can help super-heavyweight wrestlers avoid dehydration-induced performance declines. Ensuring proper sodium regulation and adjustment of fluid intake is critical in the final stages before competition [28].

This study also challenges conventional estimates of minimal wrestling weight (MWW) by demonstrating that 13% body fat is a more realistic threshold for super-heavyweight wrestlers, as opposed to the 5% BF suggested by the American College of Sports Medicine [17,49]. Given the unique physiological requirements of heavy and super-heavyweight combat athletes, coaches should reconsider rigid MWW standards and adapt them to practical observations and athlete-specific metrics.

Finally, the findings encourage coaches to take a holistic approach to body mass management, considering both physiological and psychological factors. Although this study did not directly assess mental resilience, the athlete’s high motivation to achieve a fifth Olympic gold medal undoubtedly contributed to his ability to withstand the physical and strategic demands of body mass reduction. Future coaching strategies should integrate mental conditioning alongside physiological monitoring to ensure optimal preparation and peak performance in competition [60].

## 5. Conclusions

This study makes a valuable contribution to sport science by presenting a detailed anthropometric profile of an Olympic champion, a finding with direct implications for talent identification and athlete development in elite wrestling. Understanding the structural characteristics of a world-class competitor enables coaches and researchers to refine selection criteria and optimize training strategies aimed at achieving high performance. Moreover, the study underscores the practical value of non-traditional methods for assessing and managing body mass in super-heavyweight wrestlers. Anthropometric fractionation, corrected girths, and bioelectrical impedance vector analysis (BIVA) emerge as key tools for precise athlete monitoring. While fractionation distinguishes between body compartments to guide body mass reduction without compromising functional muscle mass, corrected girths offer insights into muscle structure in performance-relevant areas. BIVA, in turn, provides information on cellular integrity and fluid balance, ensuring that weight loss strategies do not impair metabolic function. For coaches and nutritionists, integrating these techniques into training planning allows for more accurate adjustments and safeguards the athlete’s competitive capacity. Altogether, the combination of benchmark anthropometric data from an elite wrestler with advanced monitoring techniques offers a robust framework for athlete selection, performance optimization, and individualized intervention design.

## Figures and Tables

**Table 1:** Body composition corrected girths and anthropometric indices over time.

Variables	Eval 1 (9 January 24)	Eval 2 (6 June 24)	Eval 3 (11 July 24)	Change 1→2 (%)	Change 2→3 (%)	Change 1→3 (%)
**Body mass (kg)**	150	147.5	138.5	−1.6	−6.1	−7.6
**Body composition**						
Adipose mass (kg)	37.1	34.3	30.8	−7.5	−10.2	−17.0
Adipose (%)	24.1	22.9	21.4	−5.0	−6.6	−11.2
Muscle mass (kg)	77.4	76.7	75	−0.9	−2.2	−3.1
Muscle (%)	50.3	51.1	52.2	1.6	2.2	3.8
Bone mass (kg)	15.7	15.7	15.7	0.0	0.0	0.0
Bone (%)	10.2	10.5	10.9	2.9	3.8	6.9
Residual mass (kg)	17.9	17.6	16.7	−1.7	−5.1	−6.7
Residual (%)	11.6	11.7	11.6	0.9	−0.9	0.0
Skin mass (kg)	5.8	5.7	5.6	−1.7	−1.8	−3.4
Skin (%)	3.8	3.8	3.9	0.0	2.6	2.6
Body Fat Lohman (kg)	34.4	32.2	27.0	−6.4	−16.1	−27.4
Body Fat Lohman (%)	22.2	21.8	19.5	−1.8	−10.6	−13.8
FFM Lohman (kg)	115.6	115.3	111.5	−0.25	−3.29	−3.54
**Somatotype**						
Endomorphy	4.9	4.3	4.3	−12.2	0.0	−12.2
Mesomorphy	10.4	10.4	10.1	0.0	−2.9	−2.9
Ectomorphy	0.1	0.1	0.1	0.0	0.0	0.0
X	−4.8	−4.2	−4.2	−12.5	0.0	−12.5
Y	15.9	16.4	15.8	3.1	−3.7	−0.6
**Corrected girths (cm)**						
Arm	38.5	38.7	38.2	0.5	−1.3	−0.8
Chest	121.6	121	118.2	−0.5	−2.3	−2.8
Thigh	77.2	73.6	76	−4.7	3.3	−1.6
Calf	42	44.2	42.4	5.2	−4.1	1.0
**Indexes**						
BMI (kg/m^2^)	39.7	39	36.6	−1.8	−6.2	−7.8
AKS (g/cm^3^)	1.55	1.57	1.52	1.3	−3.2	−1.9
WHR	0.6	0.6	0.5	0.0	−16.7	−16.7
HBBI	23.4	23.4	23.4	0.0	0.0	0.0
HBiiL	5.4	5.4	5.4	0.0	0.0	0.0
BBI	1.3	1.3	1.3	0.0	0.0	0.0
MBR	4.9	4.9	4.8	0.0	−2.0	−2.0
AMR	0.5	0.4	0.4	−20.0	0.0	−20.0
Cormic index	51.9	51.9	51.9	0.0	0.0	0.0
Thoracic index	68.2	68.2	68.2	0.0	0.0	0.0
**Other**						
∑6SKF (mm) †	128	115.2	99.6	−10.0	−13.5	−22.2
∑4CG (mm) ‡	316.5	315	311.8	−0.5	−1.0	−1.5

FFM, fat-free mass; BMI, body mass index; WHR, waist-to-height ratio; HBBI, height-to-biacromial breadth index; HBiiL, height-to-biiliocristal breadth index; BBI, biacromial–biiliocristal breadth index; MBR, muscle–bone ratio; AMR, adipose–muscular ratio; ∑6SKF, sum of 6 skinfolds; ∑4CG, sum of 4 correct girths. † Sum of triceps, subscapular, supraspinal, abdominal, front thigh, and medial calf skinfold thicknesses. ‡ Sum of correct girths: Arm, chest, thigh, and calf.

**Table 2:** Evolutionary assessment of body composition from Bioelectrical Impedance Analysis (BIA): Estimates of the different levels of body composition studied (molecular and cellular).

Variables	Eval 1	Eval 2	Eval 3	Change 1→2 (%)	Change 2→3 (%)	Change 1→3 (%)
	(9 January 4)	(6 June 4)	(11 July 24)			
**Cellular level**						
PA (°)	6.1	5.6	5.6	8.2	0	8.2
ECW (L)	31.8	34.3	34.4	7.9	0.3	8.2
ECW (%)	21	23.5	24.7	11.9	5.1	17.6
ICW (L)	39.1	47.8	46.9	22.3	−1.9	19.9
ICW (%)	29.3	31.6	34.1	7.8	7.9	16.4
ICW/ECW	1.22	1.39	1.36	13.9	−2.2	11.5
ECW/ICW	0.81	0.72	0.73	−0.11	0.01	−0.09
Xc (Ω)	40.9	32.4	32.4	−20.8	0	−20.8
**Molecular level**						
TBW (L)	70.9	82.1	81.3	15.8	−1	14.7
TBW (%)	50.3	50.3	58.8	0	16.9	16.9
R (Ω)	380.7	342.4	333.1	−10.1	−2.7	−11.5
FMI (kg/Ht^2^)	12.68	9.85	7.59	−22.3	−22.9	−40.1
FFMI (kg/Ht^2^)	27.01	29.19	27.45	8.1	6	−1.6

PA, phase angle; TBW, total body water; ECW, extracellular water; ICW, intracellular water; FMI, fat mass index; FFMI, fat-free mass index; Xc, reactance; R, resistance.

## Data Availability

The original contributions presented in this study are included in the article. Further inquiries can be directed to the corresponding authors.

## Abbreviations

The following abbreviations are used in this manuscript:

BM	Body Mass
BIA	Body Electrical Impedance Analysis
MWW	Minimum Wrestling Weight
BF	Body Fat
FFM	Fat-Free Mass
